# Mechanisms Involved in the Modification of Textiles by Non-Equilibrium Plasma Treatment

**DOI:** 10.3390/molecules27249064

**Published:** 2022-12-19

**Authors:** Gregor Primc, Rok Zaplotnik, Alenka Vesel, Miran Mozetič

**Affiliations:** Department of Surface Engineering, Jožef Stefan Institute, 1000 Ljubljana, Slovenia

**Keywords:** non-equilibrium plasma, textiles, surface modification

## Abstract

Plasma methods are often employed for the desired wettability and soaking properties of polymeric textiles, but the exact mechanisms involved in plasma–textile interactions are yet to be discovered. This review presents the fundamentals of plasma penetration into textiles and illustrates mechanisms that lead to the appropriate surface finish of fibers inside the textile. The crucial relations are provided, and the different concepts of low-pressure and atmospheric-pressure discharges useful for the modification of textile’s properties are explained. The atmospheric-pressure plasma sustained in the form of numerous stochastical streamers will penetrate textiles of reasonable porosity, so the reactive species useful for the functionalization of fibers deep inside the textile will be created inside the textile. Low-pressure plasmas sustained at reasonable discharge power will not penetrate into the textile, so the depth of the modified textile is limited by the diffusion of reactive species. Since the charged particles neutralize on the textile surface, the neutral species will functionalize the fibers deep inside the textile when low-pressure plasma is chosen for the treatment of textiles.

## 1. Introduction

**Figure 1:**
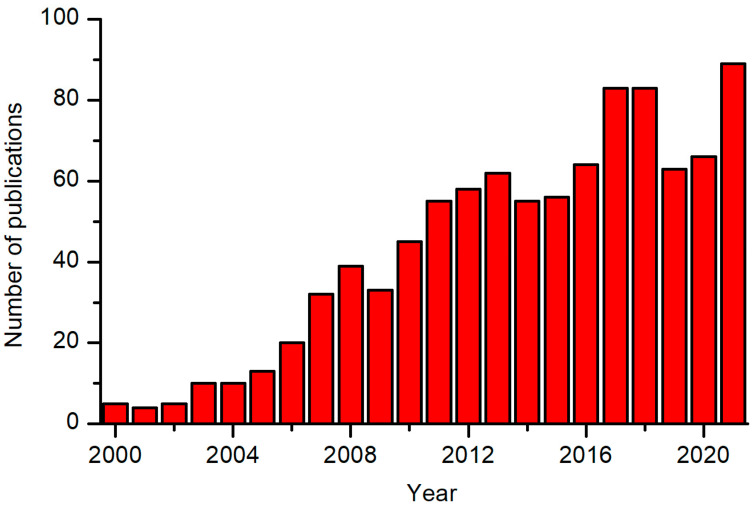
The number of publications published each year on plasma modification of textiles. The data were obtained from the Web of Science by searching with the keywords “textile” and “plasma” and “surface”.

Textiles are made from fibers of various materials, typically polymers. The properties of polymeric fibers depend on the type of materials and synthesis procedure. The ability of textiles to capture liquids depends on several parameters, and the most important is the surface wettability of the materials the fibers are made from. The surface wettability depends on the surface chemistry, particularly the polarity of surface functional groups. Most polymers contain an inadequate concentration of polar surface functional groups, so the wettability is usually below the level desired in numerous applications, particularly when attempting to bond chemically any foreign material. In cases when increased wettability is needed, the surface functional groups should be altered by grafting more polar groups. A common technique is the application of gaseous plasma. Although the technique has been used for some types of textiles on an industrial scale for decades, the scientific aspects are still a subject of investigation. Numerous scientific articles have been published on plasma modification of textiles, and the trend is shown in Figure 1. Only a few publications were published annually before 2000, and the number of publications increased by about 10-times in the past 20 years. Despite the increasing knowledge provided in these publications, the mechanisms involved in the modification of textile materials are still inadequately understood. In particular, the surface mechanisms on the atomic level are still a subject of research, even for smooth polymeric materials. The recommended theoretical papers on atomic-scale mechanisms on smooth polymer surfaces include [1,2,3], while the experimental observations on the surface kinetics were provided in [4,5]. Some review papers on plasma modification of textiles have also been published explaining the observations reported by various authors, such as [6,7,8,9,10,11,12,13,14,15,16,17]. Numerous books on various aspects of plasma treatment of textiles have been published, including a textbook intended for users of plasma technologies in the textile industry rather than experts in plasma science [18]. Despite a vast literature, the mechanisms of plasma-species penetration in fibrous materials, physical and chemical interactions with the plasma species, and the resulting surface finish of fibers deep inside the textiles are rarely explained to the level useful for scientists involved in the modification of woven and non-woven textiles with gaseous plasma. This paper intends to provide scientific aspects which are crucial for understanding plasma–textile interactions. Most recent papers are cited where appropriate. A reader will find prior publications in the citing literature.

## 2. Gaseous Discharges and Plasma Species

Non-equilibrium plasma is usually sustained with an appropriate gaseous discharge. A voltage source is used to create an electric field strong enough to ensure the acceleration of free electrons, which thus gain appropriate energy for ionization collisions with neutral molecules. Various discharges have been reported by different authors. Low-pressure discharges usually operate with a power generator at high frequency in the range of radiofrequency (RF) or microwave (MW), roughly between 10 kHz and 10 GHz. The use of high-frequency fields is preferred since it ensures a rather homogeneous plasma in a large volume without risking arcing. Furthermore, high-frequency discharges can be operated in the electrodeless mode, i.e., any electrode placed outside the discharge chamber. This is useful because the electrodeless configuration overcomes all problems associated with an electrode placed inside the chamber, such as sputtering [19] the cathode by energetic ions and deposition of cathode material on treated samples, and the loss of reactive plasma species by heterogeneous surface recombination [20]. Namely, the loss of atoms on metallic surfaces is usually 10–1000-times more extensive than on surfaces of inert materials such as glasses and smooth polymers [21]. 

The microwave discharges (frequency between about 1 and 10 GHz) always operate in the electrodeless mode. The electric field provided by an MW generator will not penetrate deep into plasma because of the skin effect [22]. Namely, non-equilibrium gaseous plasma is a weakly or moderately conductive medium with an Ohmic character of the impedance, so the penetration depth of the electric field is limited to the skin layer between the dielectric discharge chamber and the gaseous plasma [23]. The higher the frequency, the thinner the skin layer. A textile sample is placed inside the plasma, so there is no electric field nearby except a small field due to the difference between the plasma and textile surface potentials, which creates a negative potential on the textile surface of about 10 V. As a consequence, no highly energetic gaseous specie will interact with the textile when an MW discharge is used.

The radiofrequency discharges operate either in the electrodeless mode or with an electrode mounted inside a metallic chamber, which is typically grounded [24]. The electrodeless mode is further divided into the E and H modes [25,26]. In the first case, the samples are usually at the floating potential, the same as if plasma is sustained with MW discharges. In the case of an electrode inside the chamber, the sputtering may be suppressed by careful selection of processing parameters but cannot be avoided, so at least a very thin metallic film (or clusters of metallic atoms) will deposit on the textile surface. Furthermore, extensive loss of molecular radicals will occur on the metallic surface, so the density of radicals, such as free atoms, will be orders of magnitude smaller than in cases plasma is sustained with an electrodeless RF discharge. Regardless of the excitation mechanisms, the penetration depth of the RF field inside plasma depends on plasma (not discharge) parameters and increases with decreasing frequency of the power source and with decreasing density of charged particles. This is a somewhat simplified illustration useful for scientists involved in the plasma treatment of textiles. Details are still a subject of scientific research [27,28].

**Figure 2:**
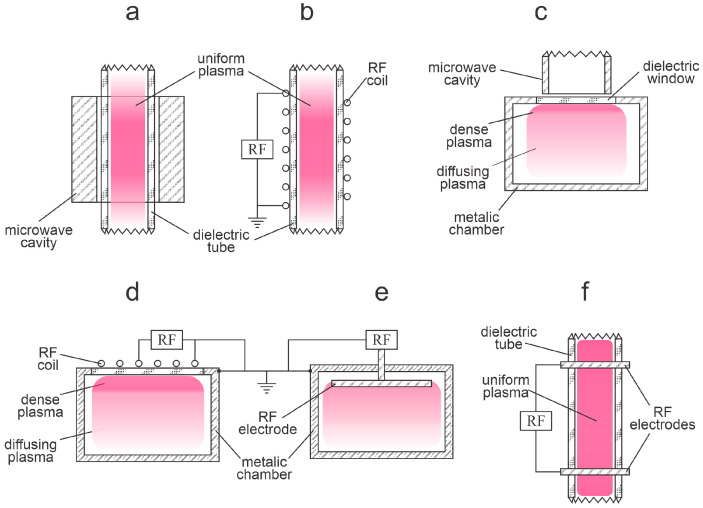
Examples of low-pressure high-frequency discharges. (**a**) Microwave in a dielectric tube, (**b**) inductive cylindrical RF in H-mode, (**c**) microwave in a metallic chamber, (**d**) inductive RF in a metallic chamber, (**e**) classical capacitive RF, and (**f**) electrodeless capacitive RF.

**Figure 3:**
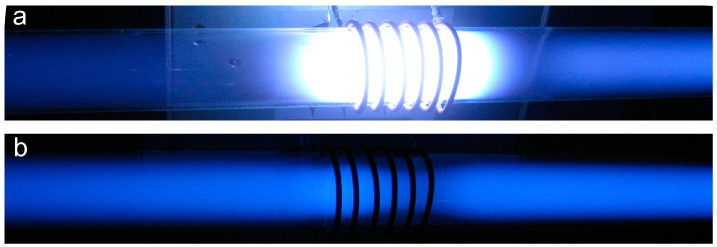
Photos of inductively coupled RF discharge in two distinguished modes: (**a**) H-mode (almost pure inductive character of the impedance) and (**b**) E-mode (with predominant capacitive component).

**Figure 4:**
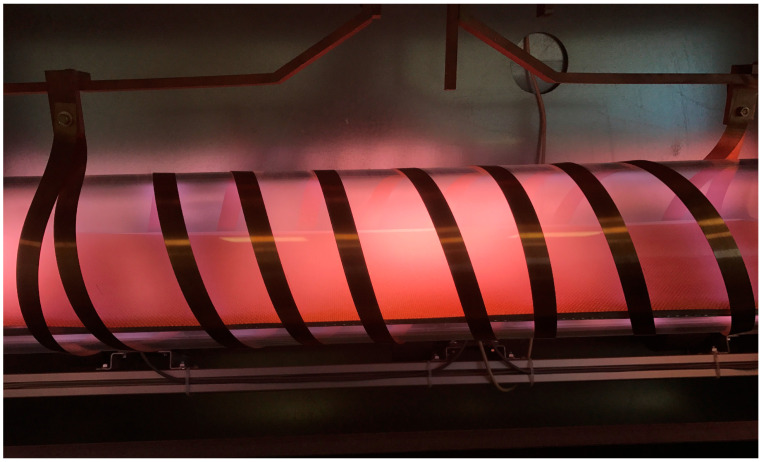
A photo of a textile in a plasma reactor powered with a rather low-power RF discharge.

As explained above, the charged particles are accelerated only in the volume of the significant electric field, which is always limited. In the rest of the discharge chamber, there will be diffusing plasma—plasma of practically zero electric field but rich in plasma species because of the presence of rather energetic electrons. The volume of a large electric field in a low-pressure gaseous plasma is often limited to a sheath next to the electrode, which may or may not be inserted in the discharge chamber. The loss of electrons’ energy in the gas phase at low pressure is rather weak because of the low collision frequency, so practically all loss mechanisms take place on the surfaces. The same applies to other reactive plasma species (ions, atoms in the ground and metastable states, and metastable molecules). Typical examples of high-frequency low-pressure discharges and resulting plasmas are shown in Figure 2. Photos of inductively coupled RF plasma in the H and E modes are shown in Figure 3. A photo of a textile in a capacitive electrodeless plasma is shown in Figure 4.

Figure 3 shows the drastic difference between the intense plasma sustained by the RF discharge in the H-mode and the weakly ionized plasma sustained in the same tube and the same RF generator but in the E-mode. In fact, the plasma luminosity in the H mode is often several orders of magnitude larger than in the E-mode, and the reason is in excellent coupling between the generator and plasma electrons [29]. The electron density is much larger in the H-mode, but the density of neutral reactive species in both modes is comparable and much larger than the density of charged particles, usually above 1020 m^−3^ [30]. The low-pressure plasma in the H-mode is, therefore, useful in cases when the material should be processed with charged particles and radiation but may not be suitable in cases when the neutral reactive species will do the job. Figure 3b and Figure 4 also indicate a rather uniform plasma in a large volume when the discharge is coupled in the E-mode, while the H-mode RF plasmas are concentrated to a rather small volume, as also illustrated in Figure 1.

RF and MW discharges can also be used for sustaining plasma at atmospheric pressure, but the plasma volume will be limited to a small volume where the highest electric field appears. Namely, the diffusing plasma cannot expand to a large volume at atmospheric pressure due to numerous collisions of free electrons with gaseous molecules or atoms. The electrons lose their energy at inelastic (and, to a much lower extent, at elastic) collisions with molecules, so they do not diffuse far from the volume of a large electric field when the pressure is around the atmospheric (1 bar). Therefore, large spatial gradients in the density of charged particles are typical for atmospheric-pressure plasma sustained with high-frequency discharges, for example, at the industrial RF frequency of 13.56 MHz or MW frequency of 2.45 GHz. In addition, such discharges operate in the continuous mode even at atmospheric pressure, and the majority of discharge power is spent on heating the gas (increasing gas temperature), so the application of such high-frequency plasmas is limited at a pressure below, say, 30 mbar.

Low-frequency RF discharges or pulsed direct-current (DC) discharges are preferred plasma sources at atmospheric pressure. In such cases, plasma is not sustained in the continuous mode but rather in the form of stochastically distributed streamers [31]. The streamers are actually ionization wavefronts [32] born on an electrode and move away from the electrode at the speed of roughly 10^4^ m/s [33,34]. They leave non-equilibrium gaseous plasma beyond them, but the plasma species are quickly thermalized at atmospheric pressure, so their density at a given position is marginal before the next streamer appears in the same volume unless plasma is sustained in high-purity noble gas [35]. Streamers are very narrow and short in duration, typically of the order of µm and µs, respectively. The maximal density of charged particles in a streamer is large, often above 10^20^ m^−3^ [36]. In the limiting case of repetitively pulsed discharges sustained by very fast switchers (switching time of the order of ns), the streamers form bullets that may propagate far from the electrode, sometimes close to 1 m [37].

**Figure 5:**
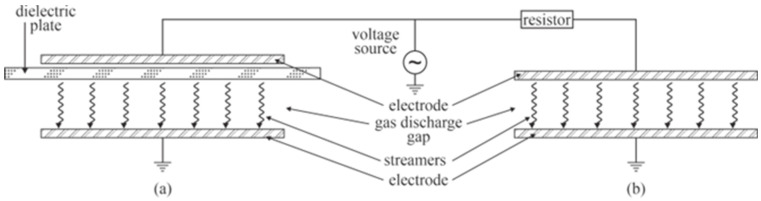
Schematic of the DBD (**a**) and the corona (**b**) discharges. The voltage source could be low-frequency RF, alternative current (AC), direct current (DC), or pulsed voltage source.

Low-frequency and pulsed discharges at atmospheric pressure come in various modes. The scientific literature usually distinguishes two types of discharge: dielectric-barrier discharges (DBD) and corona discharges. DBD employs a dielectric barrier on an electrode to limit the current of a specific streamer, whereas corona employs a resistor mounted in series with the plasma. The electrical current in a particular streamer sustained with the DBD is limited by the available charge that could be transferred from the dielectric surface to the counter electrode. In the case of the corona, the voltage drop will appear on the resistor rather than on the gas gap when the electric current flows, so the voltage across the gas gap will drop below the value useful for sustaining the gaseous discharge during the current pulse. When the electric current diminishes, the entire available voltage (the source voltage) appears again across the gas gap, so another streamer appears. A schematic of the DBD and corona discharges are shown in Figure 5.

**Figure 6:**
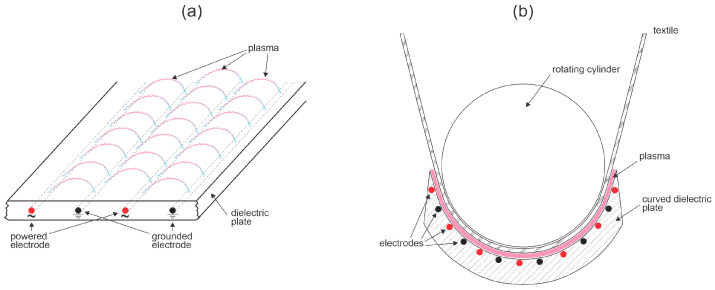
Schematic of the surface discharges sustained in the DBD mode (**a**) and a feasible solution to assure a constant distance between the textile and the dielectric plate for uniform treatment of textile surfaces (**b**).

The illustrations in Figure 6 represent the basic principles of such discharges. In practice, there are various configurations. When atmospheric-pressure plasma is used, textiles are usually processed in the continuous mode to benefit from the automatization of the plasma processing. The speed of the textile running through plasma is often close to 1 m/s, so special configurations should be used. One of them is application of surface or coplanar discharges [38,39,40]. In the case of surface discharges, the electrodes are incorporated as conductive wires into a dielectric plate as shown schematically in Figure 6a. Streamers therefore conduct the electric current on the surface of the dielectric material. The reason for such surface streamers is that the breaking voltage for air at atmospheric pressure is much smaller than for the dielectric plate providing the plate is made from a material of very high resistance, for example, glass or some types of ceramics. The streamers will appear stochastically along wires, as in the classical DBD configuration shown in Figure 6, but will be dense so the entire surface of the dielectric plate will be covered by luminous plasma. Plasma will expand perhaps 1 mm from the surface, so the distance between the textile and the dielectric plate is a crucial parameter governing the surface finish of textiles treated with plasma sustained by surface discharges. A feasible solution is shown in Figure 6b. The system shown in Figure 6 operates well as long as the impedance of any material between the electrodes is much larger than the sum of impedances of dielectric material above the electrodes and gas above the dielectric plate (Figure 6a). This requirement is not trivial and dictates innovative solutions of coupling the power supply to the wires inside the dielectric plate. The connecting wires (not shown in Figure 6a) should be placed inside a perfectly insulating liquid that is cooled to prevent overheating. Namely, a significant fraction of the discharge power is spent on heating the dielectric plate.

Corona discharges useful for treatment in the continuous mode are usually sustained along one or more electrodes mounted next to a grounded roll. The textile runs in the space between the powered electrode and the grounded roll. The preferred embodiment is not much different from the one illustrated in Figure 6b, except that the corona discharge sustains plasma instead of the coplanar DBD. In both cases, it is vital to keep the distance between the powered electrodes and the textiles as constant as possible to enable uniform surface treatment.

Regardless of the type of discharge used for sustaining gaseous plasma, the following species are found in gaseous plasma:

- Free electrons and positively charged molecules or atoms;
- Neutral molecular radicals, including atoms, in the ground electronic state;
- Metastable atoms and molecules in excited electronic states;
- Radiation in the range from infrared (IR) through visible (vis) and ultraviolet (UV) to vacuum ultraviolet (VUV);
- Negatively charged ions (important in the cases plasma is sustained in electronegative gases).

The fluxes of these species on the surface of any samples, including textiles, depend enormously on the type of discharge used for sustaining gaseous plasma. Typical numerical values will be disclosed in the following text.

## 3. Penetration of Gaseous Plasma in Textiles

**Figure 7:**
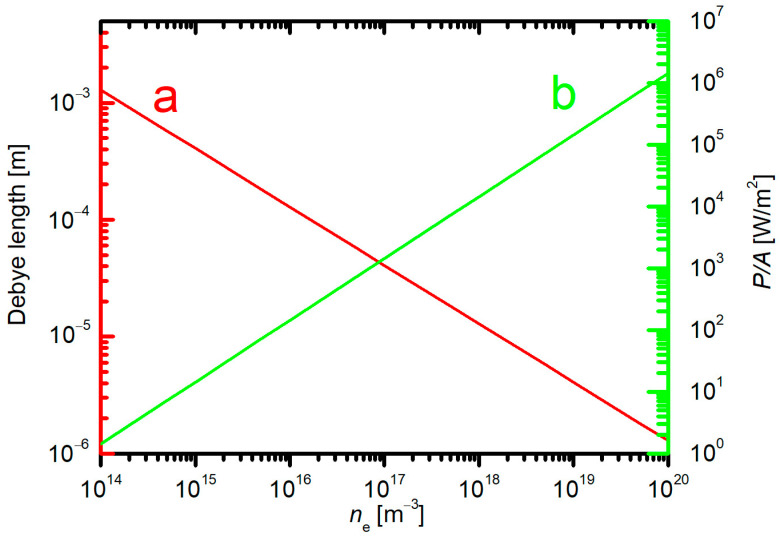
The Debye length (**a**) and the power dissipated on a surface facing a continuous plasma (**b**) versus the electron density at *kT*_e_ = 3 eV.

Usually, uniform treatment of fibers within a textile sample is preferred to the localized treatment of fibers stretching from a textile surface, so the penetration depth of species capable of modifying the fibers is important information. By definition, plasma is at least partially ionized gas with a density of charged particles large enough to form a space charge of linear dimension larger than the distance between two pieces of solid material (usually electrodes). The space charge is absolutely necessary for sustaining gaseous plasma; otherwise, the electrons would escape to the surfaces and will thus no longer be able to sustain plasma. The voltage is screened by the space charge over the distance with a characteristic thickness of the Debye length, which is defined as

(1) $$\lambda_{D}=\sqrt{\frac{\varepsilon_{0}k_{B}T_{e}}{e_{o}^{2}n_{e}}}.$$

where *T*_e_ is the electron temperature, *k*_B_ is the Boltzmann constant, *ε*_o_ is vacuum dielectric permeability (*ε*_o_ = 8.85 × 10^−12^ F/m), *e*_0_ is the elementary charge (1.6 × 10^−19^ As), and *n*_e_ is the electron density. The Debye length is proportional to the square root of the electron temperature. The electron temperature in gaseous plasma useful for the treatment of textiles does not span over a broad range; a typical value is between 10,000 and 100,000 K, which corresponds to the average electron energy of roughly between 1 and 10 eV. On the contrary, the Debye length is inversely proportional to the square root of the electron density, which depends significantly on the type of discharge and other discharge parameters and may be anywhere between about 10^14^ and 10^20^ m^−3^. Obviously, the Debye length in plasma of electron temperature a few 10,000 K assumes any value between about 1 mm and 1 µm for low and high electron density, respectively. The Debye length versus the electron density is plotted in Figure 7a.

Low-pressure plasmas will have electron densities between about 10^15^ m^−3^ (this value is typical for diffusing plasma) and about 10^18^ m^−3^ (typical for powerful inductively coupled plasma in the H-mode or a microwave plasma sustained in a rather small discharge tube). The electron density of 10^18^ m^−3^ corresponds to the Debye length of 10 µm (Figure 7a). Obviously, the low-pressure plasma is not likely to penetrate deep into the textile unless a powerful discharge sustains it. A plasma of high density of charged particles will cause significant heating of an immersed material even if the material is kept at a floating potential. The geometrical surface of a plasma-facing material is subjected to heating by ions, which will neutralize on the surface and will bombard it with the kinetic energy gained by crossing the sheath between the non-perturbed plasma and the sample [41]. The power dissipated on the surface of any material (including textile) due to the interaction with positively charged ions is

(2) $$P_{\mathrm{charged}}{=n}_{\mathrm{ion}}\cdot v_{\mathrm{Bohm}}\cdot E_{\mathrm{ion}}\cdot A.$$

where *v*_Bohm_ is Bohm velocity ($v_{\mathrm{Bohm}}=\sqrt{k_{B}\cdot T_{e}/m_{i}})$, *m*_i_ is the mass of the positively charged ions, *n*_ion_ is positive ion density in the bulk plasma, *E*_ion_ is the sum of ionization energy and the kinetic energy of an ion, and *A* is the geometrical surface area. The dissipated power per surface area is plotted versus the electron density in Figure 7b. A plasma of the Debye length 10 µm (electron density 10^18^ m^−3^) will cause heating at a power as large as 10^4^ W/m^2^. Obviously, either the density of charged power is too low to enable plasma penetration inside the textile, or it is too high to avoid overheating of the textile surface. From the plasma-penetration point of view, the low-pressure plasmas do not seem to be useful for the treatment of textiles unless the treatment time is extremely short. The benefits of using low-pressure plasmas will be explained below.

**Figure 8:**
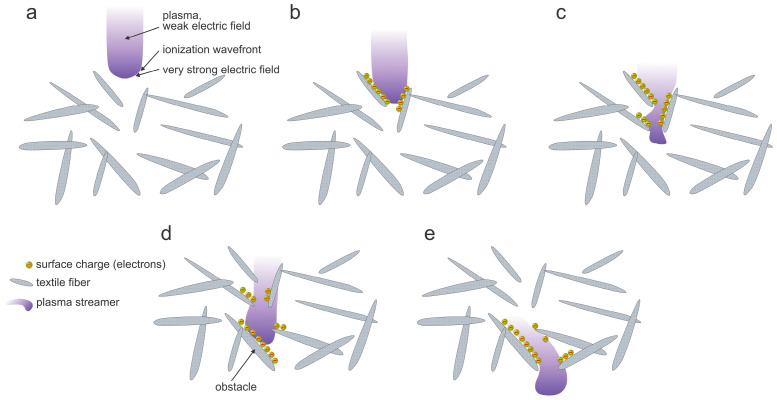
Illustration of a streamer penetration inside the textile and propagation through textile fibers from (**a**) to (**e**).

The density of charged particles within a streamer of an atmospheric pressure discharge is often much larger than in continuous plasma (for example, plasma sustained at low pressure). From this point of view, atmospheric-pressure plasma sustained by DBD or corona has advantages over continuous plasmas. The high plasma density will ensure for penetration of streamers in volume between the fibers. The schematic of the penetration of a streamer through the textile is illustrated in Figure 8. A streamer touches the textile surface (Figure 8a). The electrons are much faster than the ions (the velocity is $v=\sqrt{2W/m})$, where *W* is the kinetic energy and *m* the mass of a particle, so they charge the neighboring fibers negatively (Figure 8b). The negative surface charge will suppress the loss of electrons on surfaces, so the electron density within the streamer will remain large enough to sustain the plasma, which will propagate inside the textile (Figure 8c). Any obstacle on the streamer’s way will be charged negatively (Figure 8d), thus re-directing further propagation (Figure 8e), so finally the streamer may actually penetrate the textile providing the electron density in the original streamer (Figure 8a) is large enough. The streamer will leave behind a partially dissociated gas, and the molecular radicals (in particular free atoms) will interact chemically with the fibers’ surfaces. A benefit of such streamers propagating within porous material such as textile is that the molecular radicals are formed inside the textile (by electron-impact dissociation), so they can interact with fibers deep in the textile despite the short shelf-time typical for atmospheric-pressure plasmas. The drawback is non-uniform treatment because the streamers are stochastically distributed over the textile surface. The wettability, however, is a macroscopic parameter, so even a relatively small fraction of fibers inside the textile will ensure improved wettable and/or soaking properties. The effect is yet to be elaborated on in scientific literature, so the simplified explanation provided in this paragraph should be taken just as an illustration of the penetration of plasma streamers through textiles. The most important difference between streamers and continuous plasma is that the streamers will not destroy the textile by overheating because they carry negligible energy as compared to the flux of plasma species in the continuous mode.

The surface discharges powered by generators of frequency up to about a few 10 kHz illustrated in Figure 6 also consist of streamers, but those streamers will not penetrate the textile because they propagate on the dielectric’s surface. The streamers of surface discharges will thus only touch the textile surface, which is beneficial for processing thin textiles but may not always be useful for reasonably uniform functionalization of thicker fabrics.

Differently to streamers, continuous plasma of reasonable density of charged particles will not penetrate the porous material because the Debye length is larger than the distance between neighboring fibers in a textile. Instead, the movement of reactive plasma particles will be governed by diffusion. One exception is the penetration of radiation. The interested photon energy is beyond the threshold for breaking bonds in polymer materials, i.e., above several eV, i.e., the UV and VUV range of wavelengths. The absorption depth of photons depends on the type of polymer and concentration of impurities that act as absorption sites. The absorption coefficient is a complex function of the photon energy even for pristine polymers [42], let alone textiles made from finite-purity materials. As a rule of thumb, the penetration depth decreases with increasing photon energy and becomes only 0.1 µm or less when the photon energy is above, say, 8 eV [43]. Such energetic radiation will, of course, penetrate the gaps between the fibers but will be absorbed into the relatively thin surface film of a fiber. If the textile porosity is large, the radiation will penetrate deep into the textile and break bonds in the surface film of polymer fibers. The dangling bonds may be occupied with gaseous molecules, so the functionalization with new functional groups may occur upon exposure of textiles to (V)UV radiation in the presence of a reactive gas such as oxygen.

**Figure 9:**
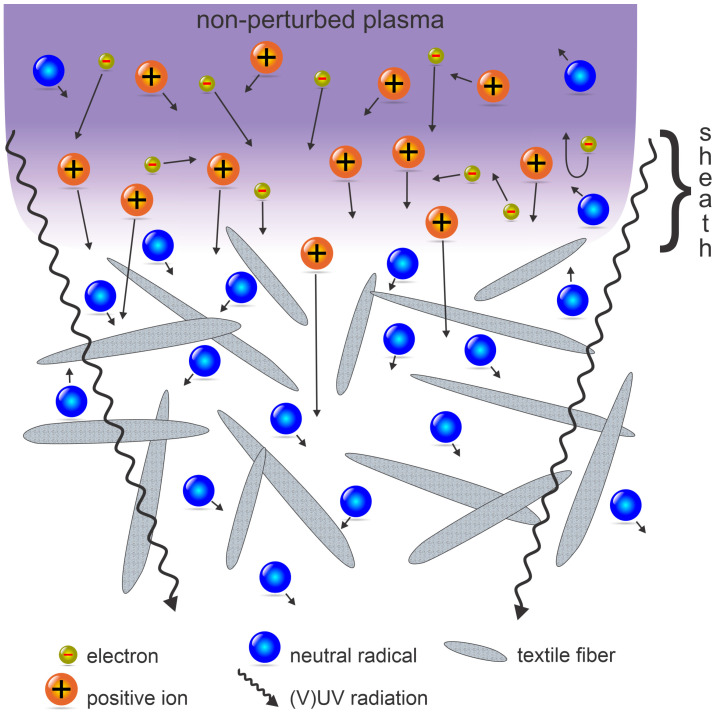
An illustration of the penetration path of VUV and UV radiation, charged particles, metastables, and radicals in textiles.

The penetration depth of particles such as charged particles, metastables, and radicals in the textile treated with a continuous plasma will depend predominantly on the surface loss coefficients. As already mentioned and illustrated in Figure 8b, the electrons are fast as compared to any other plasma particle, so they will be the first to be lost by attachment to the fiber’s surface. The positively charged ions will be attracted by the negative surface charge and will also be lost quickly because the surface neutralization efficiency is practically 100%. Negatively charged ions will be reflected upon entering the sheath, so they cannot reach the textile surface unless in cases of intentional RF biasing, which is beyond the scope of this text. On the other hand, metastables and radicals may suffer numerous collisions with the polymer surface before they finally relax, recombine, or are lost by chemical bonding to the polymer surface. The penetration depth of various plasma species in cases of streamer-free discharges (i.e., continuous plasma) is illustrated in Figure 9.

## 4. Surface Modifications Caused by Plasma Species

Once the penetration depths are known, it is possible to provide an illustration of the textile modifications upon treatment with gaseous plasma. Obviously, the inhomogeneous treatment over the entire thickness of the textile cannot be avoided but could be suppressed by the appropriate design of the treatment device and choice of the discharge parameters. Below are listed the effects of treatment by different plasma species.

### 4.1. Electrons

As illustrated in Figure 8 and Figure 9, the electrons will cause a negative charge of the fibers on the surface (Figure 9) or even deep inside the textile (Figure 8). The negative charge will retard all but the fastest electrons arriving from the gaseous plasma toward the fibers’ surfaces. As a result, the kinetic energy of a vast majority of electrons impinging the polymer surface will be below the threshold for breaking bonds. Therefore, the electrons have little effect on the textile modifications, except in the case of RF biasing, which is beyond the scope of this text. In almost all practical cases, the effect of electrons on the surface finish of textiles is marginal.

### 4.2. Positively Charged Ions

**Figure 10:**
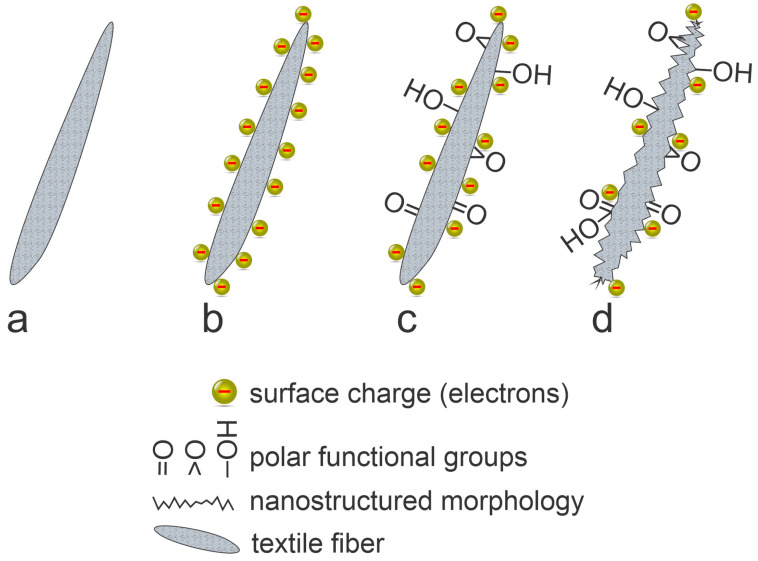
An illustration of a polymer fiber surface finishes upon interacting with positively charged oxygen ions: **a**—before interaction with plasma, **b**—immediately after turning on the plasma, **c**—after saturating the surface with polar groups, and **d**—after prolonged treatment.

If the negatively charged electrons are retarded by the surface charge, the positively charged ions are accelerated toward the surface, as illustrated in Figure 8 and Figure 9. There is always a successive negative charge on the surface of any material facing plasma, so the positively charged ions will impinge surfaces full of negatively charged electrons and will neutralize. Surface neutralization is very efficient, with the probability very close to 100%. The excessive energy (the ionization energy) will be dissipated on the fiber surface, so the surface will be heated. Furthermore, the kinetic energy of positively charged ions impinging the surface will also end up heating the fibers according to Equation (2). The kinetic energy of ions (about 10 eV in the most common case when the textile is left at floating potential) is larger than the bond strength in the polymer materials (several eV) so the ions impinging the surface are likely to interact chemically with the solid material. On the other hand, the kinetic energy of positively charged ions (roughly 10 eV when the textile is at floating potential) will not cause sputtering. The effect of the interaction between the positively charged ions and the pristine polymer is surface functionalization with groups that result from chemical interaction. If the ions are O_2_^+^, O^+^, or (OH)^+^, the newly formed functional groups will be C-OH, C=O, O-C=O, C-O-C, and so on. The effect will be limited to the very surface of the fiber unless some diffusion of successive surface oxygen occurs. The surface will soon be saturated with polar functional groups. Prolonged treatment will cause oversaturation, formation of low molecular weight fragments, and desorption of such fragments from the surface. Macroscopically, these effects will cause etching. The etching will be enhanced due to the heating caused by the dissipation of the potential and kinetic energy of positively charged ions impinging the surface. The etching is rarely laterally uniform, so nanostructuring of the fibers will occur. The combination of polar surface functional groups and rich morphology on the sub-micrometer scale will cause a super-hydrophilic surface finish [44] and, thus, excellent soaking dynamics upon wetting the textile. Obviously, the surface finish will depend on the fluence of ions on the surface, the surface temperature, and the type of polymer. The effect of positively charged ions is illustrated in Figure 10.

### 4.3. Negatively Charged Ions

As already mentioned, the surfaces facing non-equilibrium gaseous plasma are biased negatively against the plasma because of the adsorption of free electrons from gaseous plasma. The negatively charged ions (relevant only for plasmas sustained in electronegative gases) will be retarded by the surface potential and be directed back to bulk plasma. Therefore, the negatively charged ions will not reach the fiber surface, so any discussion of interaction with the fibers is not necessary.

### 4.4. Metastables

Electrons of adequate energy will cause the excitation of both neutral molecules and atoms, as well as ions, into electronically excited states. The excitation occurs in the gaseous plasma where the electron density and temperature are rather large. The excited states may be resonant, meaning that they will immediately (in a time measured in nanoseconds) relax by emitting a photon. Many states, however, are metastable because of the rules of quantum physics, which prevent the relaxation by electrical dipole radiation. The lifetime of such excited states may be long enough to enable the metastables to reach the surface. On the surface, the excitation energy may be transferred to an electron bonded to the polymer surface, thus causing surface modification. The effect is yet to be elaborated because very few results on the interaction between oxygen metastables and polymer materials have been published, although plasma scientists often take them into account when explaining mechanisms in oxygen plasmas [45,46,47].

### 4.5. Molecular Radicals, including Atoms

The neutral reactive particles usually govern the surface chemistry upon treatment of polymer materials with oxygen plasma [48]. In almost all practical cases, their concentration in gaseous plasma is orders of magnitude larger than the concentration of charged particles [20]. In low-pressure plasmas, the main reason for the large concentration of neutral reactive particles is the excellent stability in the gas phase. Namely, the recombination of simple radicals like atoms to parent molecules in the gas phase requires a three-body collision. Such collisions are scarce at pressures below a few mbar. The lifetime of atoms in a properly designed experimental system is more than a millisecond [49], so the radicals may pass several meters from the source (i.e., dense plasma) [50]. The recombination in low-pressure systems takes place practically exclusively on surfaces. Unlike the neutralization of charged particles with almost 100% probability, the surface recombination of neutral radicals depends enormously on the type of materials facing plasma [51], and may be as low as 0.001 for some types of polymers [52]. The neutral reactive species will therefore diffuse in the space between the fibers of the textile and may actually cause significant chemical modification of fibers deep inside the textile, providing the loss on polymer fibers by surface recombination is marginal. The atoms are often in thermal equilibrium with the neutral gas, so their temperature will not be much over room temperature. As a consequence, the atoms will not cause heating of the textile unless a chemical or physical reaction occurs on the fiber surface. The physical reaction is surface recombination to parent molecules, while the chemical reactions cause polymer oxidation. The oxidation kinetics are complex. For example, Longo et al. [1] found as many as 20 binding sites with different adsorption energies even for pristine polymer, let alone partially oxidized material. By far the most probable interaction is the substitution of C-H with C-OH [1]. Hydroxyl groups are, therefore, the first to be formed on a polymer surface. In fact, experiments show that the surface saturation with hydroxyl groups occurs at the dose of O atoms of 10^19^ m^−2^ [5]. If the atom density next to the fiber is 10^21^ m^−3^, the saturation occurs in 0.1 ms! Much larger doses are needed for saturating the surface with other oxygen-containing functional groups, but 10^22^ m^−2^ is enough for many polymers, including fluorinated ones [4].

**Figure 11:**
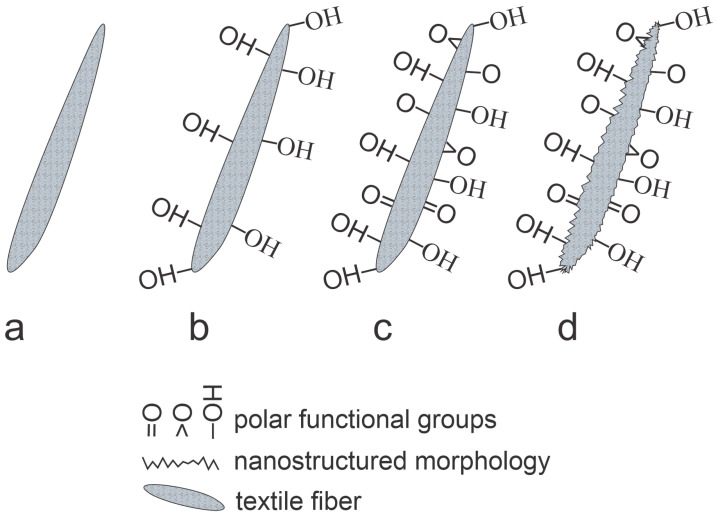
An illustration of a polymer fiber surface finishes upon interacting with neutral oxygen atoms. **a**—before interaction, **b**—after receiving the dose of about 10^19^m^−3^, **c**—after receiving the dose of about 10^22^ m^−3^, and **d**—after very large doses.

The unique advantage of using neutral radicals over positively charged ions is excellent efficiency for surface oxidation. An atom will either interact chemically with a polymer surface or will experience an elastic collision and will not cause surface heating. The heating also occurs at surface recombination, but the coefficient is low for most polymers [52], so the atoms represent the plasma species of choice when functionalization of fibers deep in the textile is desired without significant heating of the textile surface. The interaction of O atoms with a polymer fiber is shown in Figure 11. Large doses will cause etching, but the effect is marginal as compared to etching rates observed for treatment with positively charged ions.

At atmospheric pressure, the loss of radicals in the gas phase is efficient in molecular gases and less efficient in noble gases with a small admixture (typically up to about 1 vol.%) of molecular gas. That is one of the reasons for using noble gases in atmospheric-pressure plasmas. In any case, the maximal achievable atom density at atmospheric plasmas is practically the same as in low-pressure plasmas, i.e., roughly about 10^21^ m^−3^ [53]. The energy efficiency of atmospheric-pressure plasmas, especially those sustained in molecular gases such as oxygen or air, is inferior to the efficiency of low-pressure plasmas sustained in dielectric tubes by electrodeless discharges because of the extensive recombination of atoms to parent molecules at three-body collisions at atmospheric pressure.

## 5. Conclusions

An insight into the plasma techniques for the functionalization of textiles from the perspective of plasma science was presented. Plasma sustained in pure noble gases does not contain chemically reactive particles but is a source of VUV and UV radiation which breaks bonds in the polymer material and thus provides dangling bonds to be occupied upon exposure to reactive gases. The efficiency is rather inadequate because the photons are not absorbed in the very-surface film useful for functionalization with desired surface functional groups but rather in a thicker film, typically about 100 nm for very energetic photons of 8 eV and above, more for less energetic photons. The effect of other plasma species is limited to the very surface, especially when the textile is kept at a floating potential (which is true in all practical cases). The reactive plasma particles are predominantly formed at an impact with an energetic electron in gaseous plasma, but the loss will be in the gas phase at elevated pressure (atmospheric and above), and on surfaces at low pressure (a few mbar and below). In between atmospheric and low pressure, there is a range of pressures rarely tackled by scientists or users of plasma technologies. The energy dissipated at the neutralization of charged particles and the recombination of radicals, including atoms, to stable molecules will cause heating. Gaseous molecules will be heated in atmospheric-pressure plasmas, while in low pressure, the surfaces will be heated. Low-pressure plasmas are usually sustained in the continuous mode and are rich in neutral reactive particles that are useful for chemical interaction with polymer surfaces. While the atmospheric-pressure plasmas can be sustained in the continuous mode, they are not very useful for the treatment of textiles due to the high-power density needed for sustaining the continuous plasma at high pressure. From this aspect, it is advisable to use atmospheric plasmas, which operate in streamers—current pulses of duration of the order of µs occurring stochastically in the discharge gap. The streamers are actually ionization wavefronts progressing from the powered electrode at a speed of roughly 10^4^ m/s. The ionization wavefronts leave behind non-equilibrium gaseous plasma, which is of short duration because of numerous collisions which lead to the gas thermalization and thus the loss of chemically reactive particles useful for surface functionalization of polymers. An advantage that makes the atmospheric-pressure plasmas attractive for the treatment of textiles is the ability to sustain plasma even inside the textile, in the space between the fibers, providing the electron density within the streamer is large enough so that the space charge can suppress their loss on the surfaces. By using streamers of high electron density, it is possible to obtain the desired surface finish of the entire textile despite the localized character of the streamers. On the contrary, low-pressure plasmas assure a large density of molecular radicals, in particular atoms, which may diffuse in the space between the fibers and cause surface chemistry even on fibers rather deep in the textiles. The charged particles will always cause significant heating of the materials facing them, so the plasma of a large density of charged particles may cause overheating of the fibers on the textile surface while leaving the fibers deep inside the textile inadequately treated.

## Data Availability

Not applicable.

## Sample Availability

Samples of the compounds are available from the authors.
